# Measures for preventing norovirus outbreaks on campus in economically underdeveloped areas and countries: evidence from rural areas in Western China

**DOI:** 10.3389/fpubh.2024.1406133

**Published:** 2024-06-04

**Authors:** Huali Xiong, Fengxun Ma, Dayi Tang, Daiqiang Liu

**Affiliations:** ^1^Department of Public Health, Health Commission of Rongchang District, Chongqing, China; ^2^Center for Mental Health of Rongchang District, Chongqing, China; ^3^Department of Public Health, The People's Hospital of Rongchang District, Chongqing, China; ^4^First Clinical College, Mudanjiang Medical College, Mudanjiang, Heilongjiang, China; ^5^Department of Hospital Information, The People's Hospital of Rongchang District, Chongqing, China

**Keywords:** epidemiology study, norovirus, GII strains, outbreak, vomitus, underdeveloped areas and countries

## Abstract

**Background:**

The outbreak of norovirus represents a significant public health emergency within densely populated, impoverished, and underdeveloped areas and countries. Our objective is to conduct an epidemiology study of a norovirus outbreak that occurred in a kindergarten located in rural western China. We aim to raise awareness and garner increased attention towards the prevention and control of norovirus, particularly in economically underdeveloped regions.

**Methods:**

Retrospective on-site epidemiological investigation results, including data on school layout, case symptoms, onset time, disposal methods and sample testing results, questionnaire surveys, and case-control study were conducted in a kindergarten to analyze the underlying causes of the norovirus outbreak.

**Results:**

A total of 15 cases were identified, with an attack rate of 44.12% (15/34). Among them, 10 cases were diagnosed through laboratory tests, and 5 cases were diagnosed clinically. Vomiting (100%, 15/15) and diarrhea (93.33%, 14/15) were the most common symptoms in the outbreak. Case control study revealed that cases who had close contact (<1 m) with the patient’s vomitus (OR = 5.500) and those who had close contact with similar patients (OR = 8.000) had significantly higher ORs compared to the control participants. The current study demonstrated that improper handling of vomitus is positively associated with norovirus outbreak. The absence of standardized disinfection protocols heightens the risk of norovirus outbreaks.

**Conclusion:**

To our knowledge, this study represents the first investigation into a norovirus outbreak in rural areas of western China. We aspire that amidst rapid economic development, a greater emphasis will be placed on the prevention and control of infectious diseases in economically underdeveloped areas and countries.

## 1 Introduction

Norovirus (NoV) is a significant pathogen that causes non-bacterial acute gastroenteritis in humans. It typically leads to outbreaks or epidemics and affect people of all ages (1, 2). Norovirus has an extremely strong infective ability, primarily due to the lack of targeted vaccines and the virus’s rapid mutation, exposure to as few as 18 viral particles can lead to norovirus infection (3). Norovirus can be transmitted through food, drinking water, and objects contaminated with norovirus, it can also spread through aerosolized particles and close contact with infected individuals (4–6). There is usually nausea, vomiting, diarrhea, and abdominal pain accompanied with norovirus infection after an incubation period of 12 to 48 h (7). Norovirus outbreaks often occur in schools, daycare facilities, and other densely populated areas (8, 9). It is estimated at least one-fifth of acute gastroenteritis cases worldwide are associated with norovirus infection (10) and approximately 677 million worldwide are infected with norovirus each year (11). It is reported that over 200,000 died because of norovirus infection, causing approximately 4.2 billion US dollars in direct economic burden and 60.3 billion US dollars in social burden (12).

Since the first report of norovirus causing a global acute diarrhea outbreak in 1996, the GII.4 genotype variant has been the dominant strain of the virus worldwide, including in China (13). However, the GII.4 strain has begun to be replaced by other genotypes in global epidemics, such as the large-scale outbreak of the GII.17 strain in Asia during the winter of 2014 (14, 15). The GII.2[P16] strain began to replace the GII.17 strain, leading to outbreaks in Asia, Europe, and America during the winter of 2016 (16). The majority of norovirus outbreaks in China are caused by the GII type, posing an increasingly serious public health problem (17–19). The epidemiological characteristics and disease burden of different genotypes of norovirus are not the same. Therefore, it is necessary to monitor the prevalence and variation of NoV strains and develop and implement targeted prevention and control strategies.

In the Chinese general population, the annual incidence rate of norovirus is 6.0% in 2015, of which the incidence rate of children under 5 years old is 15.6% (20). 1,725 norovirus infection outbreaks were reported from 2007 to 2021, most of which occurred in schools and childcare settings (1.539 cases, 89.22%), with human-to-human transmission accounting for 73.16% of infections (21). There were 1,637 cases of norovirus infection recorded in Chongqing from 2011 to 2016, of which 1,637 cases (91.98%) occurred in urban schools and nurseries, 80% of the cases involved person-to-person transmission (9). In previous studies, epidemiological features and influencing factors of Norovirus outbreaks were mainly discussed (9, 20, 21), while there has been limited survey of Norovirus outbreaks in China.

It is believed that humans are the primary source of Norovirus transmission, and close contact is a major risk factor (22). Internal transmission can occur due to high levels of exposure to the Norovirus and secondary transmission (23). It remains an area of great interest and limited data on human-to-human transmission of Norovirus. Currently, we conducted an epidemiological study of Norovirus transmission in Qingjiang town, which was located in Rongchang, Chongqing municipality, southwest of China, a region that is economically underdeveloped.

February 9, 2023, a contingency was reported to the Center for Disease Control and Prevention of Rongchang district (RCCDC) from the Qingjiang Health Center, several students in the same kindergarten were experiencing symptoms such as vomiting and abdominal pain. The affected students had already been transported to medical institutions for treatment. Subsequently, an epidemic investigation was initiated to ascertain potential sources of infection, transmission routes, and to recommend suitable control measures for economically underdeveloped regions.

## 2 Methods

### 2.1 Case definition and search

According to the Chinese guidelines for the investigation and prevention of Norovirus outbreaks (2015 Edition) (24), the following definitions applied. Suspected cases refer to instances of acute gastroenteritis observed in the kindergarten since February 6, 2023, characterized by three or more bowel movements with altered appearance within 24 h, and/or vomiting occurring two or more times within the same time frame, and/or presenting symptoms like fever and abdominal pain. Clinical diagnosis cases are those meeting the criteria for suspected cases and demonstrating epidemiological links with laboratory-diagnosed cases involved in this infectious diarrhea cluster epidemic. Laboratory diagnosis cases encompass instances where suspected cases or those diagnosed clinically test positive for fecal, anal swab, or vomit samples via nucleic acid testing or ELISA antigen testing.

### 2.2 Epidemiological investigation

#### 2.2.1 Investigation of basic information

In accordance with the self-designed questionnaire named “Norovirus infection case investigation form,” the investigation was executed by professional epidemiological personnel who compile essential information, including the case’s basic details, symptoms of the disease, onset time, and medical treatment received. Simultaneously, an on-site inspection of the kindergarten’s public environment, cafeteria, toilets, drinking water, and other hygiene conditions is conducted. Furthermore, the physical health status of all individuals is gathered through face-to-face communication.

#### 2.2.2 Investigation of the risk factors

As reported in previous studies (9, 21), the main routes of transmission of norovirus include water source, food source, people-people and hybrid. Further investigation of the risk factors including environmental hygiene (classrooms, game area, dormitory, and toilet), watering area (drinking water, water cup, cup holder, bucket, and bucket lid), kitchen (cutting board and operating table, dining plates and chopsticks, and kitchenware), cafeteria tabletop, food processing, preparation, storage, meal portioning, food passing process and tableware disinfection process in schools involved in the outbreak.

#### 2.2.3 Case-control study

Case-control study was conducted to explore the suspected exposure factors for transmission. Cases meeting the case definition of “Clinical diagnosis cases” and “laboratory diagnosis cases” were selected as the case group, and students in the same kindergarten without any clinical symptoms were selected as the control group. The risk factors in survey mainly included “Do you wash your hands before lunch at kindergarten?,” “Do you wash your hands after using the restroom at kindergarten?,” “Have you ever eaten lunch in the cafeteria at noon?,” “Do you have close contact (<1 m) with the patient’s vomitus?,” “Have you been in closely contact with similar patients?,” “Do you drink bottled water?” according to previous studies (25, 26) in China. The whole process of the survey was completed by the staff of the RCCDC. Odds ratio (OR) and its 95% confidence interval (CI) were calculated to indicate the strength of association between exposure and morbidity, if 95% CI does not contain 1 indicates that the difference in OR values is statistically significant.

### 2.3 Sample collection

#### 2.3.1 Anal swab

Anal swabs with sampling swab dipped in virus medium, inserted into the anus at 4–5 cm (2–3 cm in young children), gently rotated and wiped the rectal surface, and then placed in the sampling tube containing virus medium. Anal swabs were obtained from 13 kindergarten students and 3 staff members as part of the investigation.

#### 2.3.2 Drinking water

1.5 liter of drinking water samples were collected using sterile containers.

#### 2.3.3 Surface swab

Surface swab samples such as door handles, toys, etc. are collected from relevant places in the outbreak according to the purposes of the outbreak investigation. A swab can be moistened in virus medium and vigorously applied to the surface to be sampled, then immediately immersed in virus medium. 31 environmental surface swab samples, encompassing areas such as the school cafeteria, kitchen, toilets, game area, toys, classrooms, dormitory, and public watering area were collected.

All samples were collected by the associate chief laboratory technician of RCCDC. Specimens were kept refrigerated at −4°C in the transfer box. Samples were transported to the RCCDC within 2 h after collection and tested immediately for the purpose of identifying the presence of bacteria and viruses. No institutional review committee is required for the current study as the samples were used for epidemic investigation and diagnosis.

### 2.4 Norovirus detection

#### 2.4.1 Enrichment of norovirus using acid-treated negative ion membrane method

The collected drinking water was filtered through filter paper. 0.5 L of water samples were measured, and 10 μL of MS_2_ phage were added. The pH of the water samples was adjusted to 3.0 with hydrochloric acid, and MgCl_2_-6H_2_O was added to achieve a final concentration of 0.05 mol/L. The water samples were slowly passed through a mixed cellulose membrane using a vacuum pump, and the filter membrane was then cut up and transferred to a 50 mL sterile centrifuge tube.

Add 20 mL of TGBE buffer to the centrifuge tube, shake at 180 times/min at room temperature for 20 min to elute the virus, transfer the liquid into another 50 mL sterile centrifuge tube, and adjust the PH to 7.0. Add 5 × PEG8000/NaCl solution to the 50 mL centrifuge tube containing the eluent to achieve a final concentration of 10%, and homogenize the liquid with a vortex shaker for 60s. Leave the tube to settle the virus overnight at 4°C. The virus was centrifuged at 10,000 × g for 30 min at 4°C, the supernatant was discarded, and the precipitate was retained. The precipitate was resuspended in PBS buffer, and the viral RNA would be extracted.

#### 2.4.2 Nucleic acid extraction

In a virus transport medium, 16 anal swabs and 31 surface swabs were stirred in a vortex for 2 min to ensure the release of viruses and genetic material, and then the supernatant was collected. Total nucleic acids were extracted from a 400 μL supernatant sample by Biological Nucleic Acid Extractor Instrument (Jiangsu Bio Perfectus, SMPE-1280, China), which was complied with manufacturer’s instructions of the Consumable kit (Jiangsu Bio Perfectus, SDK60102, China).

#### 2.4.3 Real-time RT-PCR for the detection of norovirus

5 ul of nucleic acid and 20 ul of master mix were mixed together to performed real-time reverse transcription polymerase chain reaction using a real-time fluorescence PCR instrument (Thermofisher, ABI7500, United States) with Norovirus GI/GII type nucleic acid detection kit (Jiangsu Bio Perfectus, YJC50201N, China) under the following conditions: ① reverse transcription: 50°C for 10 min, 1 cycle; ③denaturation:95°C for 5 min, and 45 cycles of PCR (95°C for 10 s and 58°C for 30 s) for a total turnaround time of 70–80 min. As soon as the reaction is completed, the result is automatically saved. Based on the automatically analyzed image, adjust the “start” value, “end” value, and “threshold” value of the baseline and click “analysis” bottom to automatically obtain the result. Positive result is considered when Cycle-threshold (Ct) is less than 37 and the curve is S-shaped while negative result is considered when Ct is over 40 or undetected. GI and GII type typing detection was performed at the same time, GI type for FAM channel and GII type for VIC channel. The whole process was carried out strictly according to the manufacturer’s instructions of the instrument and the kit.

### 2.5 Statistical analysis

All epidemiologic and laboratory data were entered into Excel 2013 and categorical data was expressed as percentages (%). Descriptive analysis is used for analysis, and the calculated indicators mainly included attack rate, positive detection rate. Case control study was conducted by SPSS. OR (odds ratio) and 95% CI (confidence interval) were calculated by univariable logistic regression. SPSS version 26.0 was applied for statistical analyses and a *p* < 0.05 was considered statistical significance.

## 3 Results

### 3.1 Background

**Figure 1:**
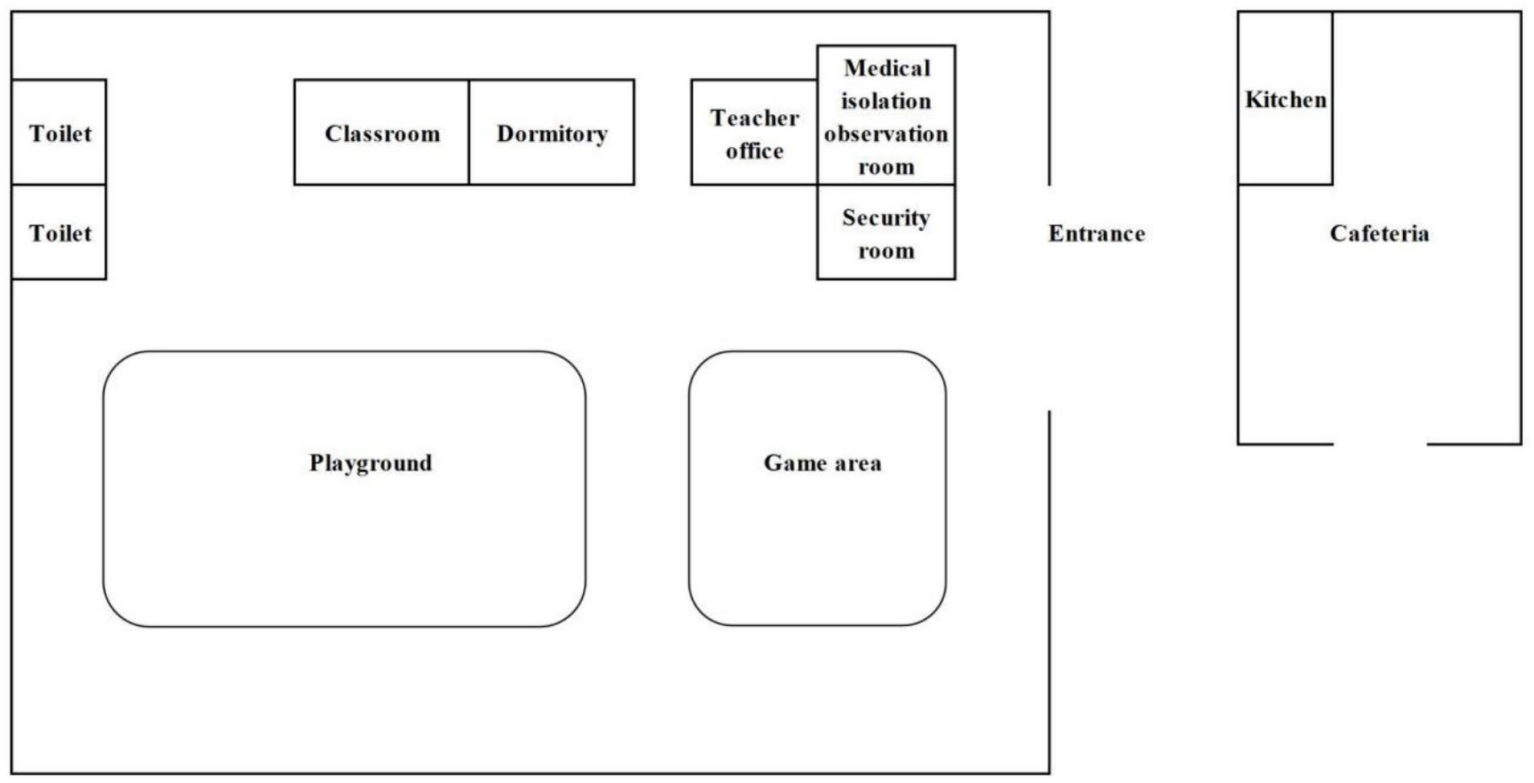
The floor plan of the kindergarten.

The kindergarten is situated in Group 5, Zhulin Village, Qingjiang Town, southwest of Rongchang District, Chongqing Municipality, southwest of China. The regional area of Qingjiang Town spans approximately 17.8 square kilometers and comprises one community and three villages, with a total population of around 14,000 residents. Notably, Zhulin Village is home to only 500 individuals. The kindergarten itself accommodates a total of 30 students, 3 teachers, and 1 chef. The school cafeteria, responsible for providing daily lunches for students and teachers, which includes a vegetable dish, a meat dish, soup and rice, is housed in a separate building outside the kindergarten. Parents are responsible for arranging breakfast and dinner for their children, as these meals are not included in the kindergarten’s services. It is pertinent to note that students are prohibited from bringing snacks to the kindergarten, and there are no mobile vendors selling food in the vicinity. Furthermore, all children and staff members drank cooled boiled water stored in insulated containers in classroom. The floor plan of the kindergarten are shown in Figure 1. (The characteristics of the exposed population are shown in Supplementary Table S1).

### 3.2 The attack rate

A total of 15 cases were identified, with an attack rate of 44.12% (15/34). Among them, 10 cases were diagnosed through laboratory tests, and 5 cases were diagnosed clinically. All affected students were kindergarten students.

### 3.3 Clinical symptoms

The clinical manifestations of 15 cases primarily included vomiting (100%, 15/15) and diarrhea (93.33%, 14/15), with some cases accompanied by nausea (66.67%, 10/15), abdominal pain (60%, 9/15), fever (6.67%, 1/15), headache (6.67%, 1/15).

### 3.4 Epidemiological characteristics

#### 3.4.1 Temporal distribution

**Figure 2:**
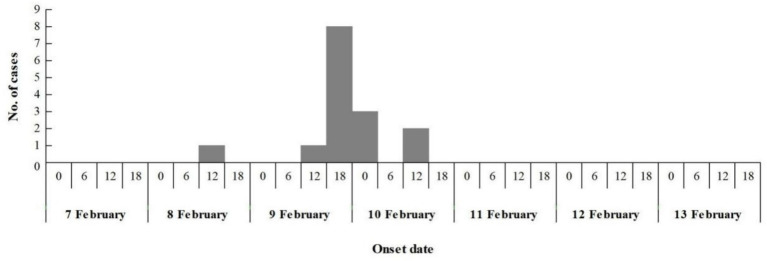
Date and time (hours) of symptom onset.

The epidemic lasted for 3 days, with 60% (9/15) of cases occurred from 6:00 to 18:00 on February 9. At 7:30 on February 8, the index case began vomiting on the way to kindergarten, and vomited again at 10:00 during class on February 8, the second case experienced vomiting in the classroom at 10:30 am on February 9, the third case commenced vomiting in the classroom at 14:30 pm on February 9. Additional 12 children fell sick between 15:00 pm on February 9 and 7:00 am on February 10, spanning a 16 h duration. No new cases were reported in the subsequent days during the possible incubation period for norovirus. This pattern suggests a point-source outbreak (Figure 2), attributable to the typical incubation period of norovirus infection, which ranges from 12 to 48 h.

#### 3.4.2 Spatial distribution

**Figure 3:**
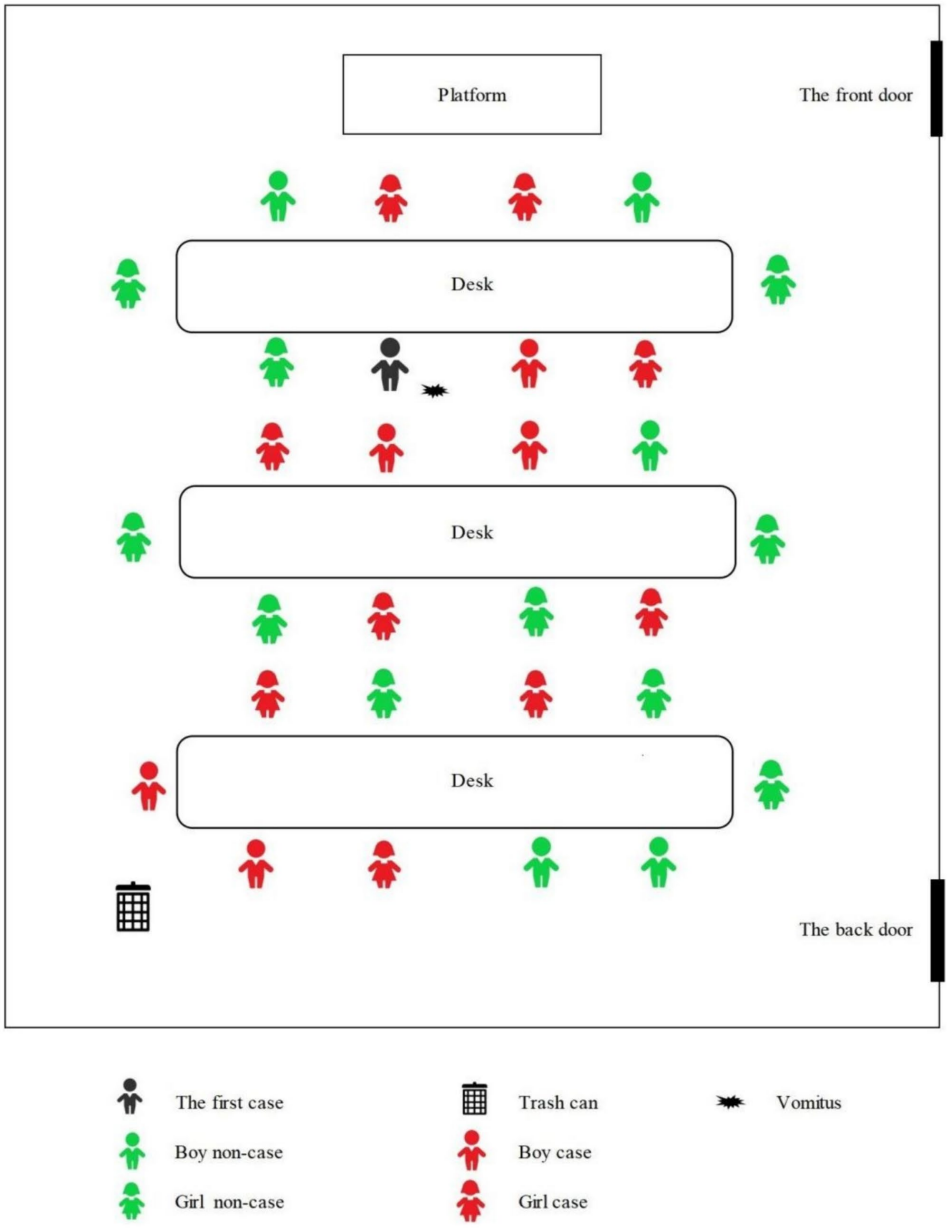
The setting arrangements of the students in kindergarten.

The seating arrangements of the students in kindergarten are shown in Figure 3. The distance between each row of desks was less than 1 meter, and the distance between the trash can and the last row of students was also not exceeded 1 meter. The black marked in Figure 3 indicated where the index case student vomited while sitting in his seat in class, after the pre-school teacher processed the vomitus, the boy continued attending class. Additionally, after class, the index case also played with a few classmates around him.

#### 3.4.3 Population distribution

Among the 15 cases, the age ranged from 3 to 6 years old, with an average of 4.6 years old. The male to female ratio is 0.66:1 (6/9).

### 3.5 Investigation of suspicious risk factors

#### 3.5.1 Food hygiene survey

The school cafeteria, situated in a separate building outside the teaching area, features a kitchen and cafeteria that are relatively independent. A single employee handled all tasks, including serving dishes, cooking, and cleaning. The hygiene standards in the kitchen and cafeteria were deemed acceptable. The school provides lunch daily, while students were responsible for their breakfast and dinner at home. There were no external food sources, and mobile vendors did not sell food on school premises. Pre-school teachers rewarded students with lollipops, with each student receiving one. All children and faculty members consumed the same food provided at school. Food processing, preparation, storage, and portioning are all done by the chef alone. After the dishes are cleaned, they are placed in a sterilizer for disinfection. The floor and table tops of the cafeteria are cleaned daily, but no sanitizer is used.

#### 3.5.2 Drinking water hygiene survey

All students and faculty members consumed cooled boiled water stored in insulated containers, and there were no alternative sources of drinking water available in kindergarten.

#### 3.5.3 Index cases and subsequent cases

At 7:30 on February 8, the index case began vomiting on the way to kindergarten and failed to inform the teacher about the incident upon arrival at school. Subsequently, at 10:00 during class, the child vomited again. During lunch, the index case exhibited symptoms of fever, mild abdominal pain, and anorexia. The teacher arranged the first case to rest in the dormitory and notified the parents to pick him up from the kindergarten at 13:00. Shortly after returning home, the symptoms exacerbated, manifesting as multiple instances of vomiting, abdominal pain, diarrhea, and watery stools. According to the teacher’s recollection, she once accompanied the child to the school’s public restroom. As per the mother’s self-description, he did not have contact with a similar case 1 week before the disease onset and did not venture outside, while during this period, coinciding with the Chinese Spring Festival, she could not accurately recall whether her children consumed any specific foods.

The second case, a girl, experienced nausea and vomiting in the classroom at 10:30 am on February 9.

The teachers implemented the same handling measures. The third case, a boy, commenced vomiting in the classroom at 14:30 pm on February 9.

#### 3.5.4 Handling of vomitus

The teacher used tissues to wipe the boy’s mouth, and the pre-school teacher employed a mop to clean the floor contaminated with vomitus; however, disinfection of the floor was not carried out. Notably, the children were not evacuated from the classroom during this process. The vomitus was deposited in the classroom trash can and later emptied into the school’s centralized trash can after the class concluded. During this outbreak, all 15 cases experienced vomiting, with 7 students vomiting at school. The cleanup of the vomitus was carried out by teachers.

### 3.6 Pathogen detection

A total of 16 human anal swab samples were collected from 13 kindergarten students and 3 staff members. The positive rate of personnel samples was 62.50% (10/16), while all 3 staff members tested negative. A total of 31 environmental surface samples and 1 water sample were collected. 17 samples were tested positive with a rate of 53.13% (17/32). The details of all samples can be seen in the Table 1. All the samples tested positive for NoV GII, while none of them were positive for NoV GI.

**Table 1:** Detection result of human and environment samples.

	Total	GII-positive^a^
**Human rectal swabs samples**	16	10
Males	8	5
Females	8	5
**Environmental samples**	32	17
**Classroom**		
Floor	3	3
Toys	2	2
Handle	1	1
Switch	1	0
Chairs	2	2
**Kitchen**		
Cutting board and operating table	1	0
Dining plates and chopsticks	1	0
Kitchenware	1	0
**Game area**		
Floor	2	0
Slide	2	1
**Toilet**		
Floor and door handles in men’s toilet	1	1
Floor and door handles in women’s toilet	1	1
Stopcock	1	0
**Dormitory**		
Floor	2	1
Bedding	2	1
Door handle	1	1
Switch	1	1
**Watering area**		
Drinking water	1	0
Water cup	2	1
Cup holder	1	0
Bucket	1	0
Bucket lid	1	0
**Cafeteria**		
Tabletop	1	1
**Total**	48	27

a All the samples tested positive for NoV GII, while none of them were positive for NoV GI.

### 3.7 Case control investigation

The case-control study on risk factors for the norovirus outbreak among students was presented in Table 2. In this study, a total of 15 infected cases and non-infected cases were interviewed. Compared with the control participants, cases who had close contact (<1 m) with the patient’s vomitus (OR = 5.500) and those who had close contact with similar patients (OR = 8.000) had significantly higher ORs compared to the control participants.

**Table 2:** Case control study of norovirus outbreak.

Variables	Case	Control	*OR* value	95% CI	*p*-value
Do you wash your hands before lunch at kindergarten?					
Yes	2	1	2.154	0.174–26.673	0.542
No	13	14			
Do you wash your hands after using the restroom at kindergarten?					
Yes	1	3	0.297	0.026–3.121	0.283
No	14	12			
Have you ever eaten lunch in the cafeteria at noon?					
Yes	15	15	—	—	—
No	0	0			
Do you have close contact (<1 m) with the patient’s vomitus?					
Yes	10	4	5.500	1.145–26.413	0.028
No	5	11			
Have you been in closely contact with similar patients?					
Yes	12	5	8.000	1.522–42.043	0.010
No	3	10			
Do you drink bottled water?					
Yes	4	3	0.615	0.264–8.009	0.666
No	11	12			

## 4 Discussion

The clinical manifestations, epidemiological investigation, and laboratory detection indicated that the outbreak was caused by Norovirus GII. The number of cases rapidly increased within the incubation period after the onset of the index case, suggesting a point source exposure pattern. However, no Norovirus was detected in environmental smears, cafeteria utensils, anal swab, or workbenches from cafeteria staff. All personnel had lunch at school, and the drinking water for the entire school was cooled boiled water, with no Norovirus detected. The case control study results revealed that close contact with patient vomitus (within 1 meter) and close contact with patients were risk factors for the outbreak. We have reason to believe that this was an outbreak of norovirus due to improper handling of vomitus by pre-school teacher. There are three reasons: Firstly, all students, pre-school teachers, and other staff consumed the same water and food, no norovirus was detected in kitchen and drinking water samples, which may help us rule out water and food as the main risk factors for outbreaks. Secondly, the epidemic curve shows a time interval of 24.5 h between the first case and other cases, indicating that cases within the cluster may have been exposed to the index case. Thirdly, after temporarily suspending classes and implementing thorough disinfection in the kindergarten, the outbreak quickly subsided. In summary, the main transmission of this epidemic was caused by the first case vomiting in the classroom, which led to environmental contamination. Other students who were exposed to this contamination fell ill within a short period and close contact resulting in the spread of the epidemic from a point source to a wider surface, ultimately leading to an outbreak.

There has not been much change in the prevalent genotype of norovirus in China. Since the 1990s, the GII type of norovirus has consistently been the dominant strain, causing outbreaks of norovirus (27, 28). During 2016, out of the 94 norovirus outbreaks in China, GII.2 was the most common genotype, accounting for 52.00% of the outbreaks (29). According to the surveillance data of the norovirus outbreak from 2016 to 2018 (19), 91.50% of outbreaks were caused by the GII, 5.50% were of the GI, and the remaining 2.50% were caused by mixed infections of GI and GII. Comprehensive network-based epidemiological surveillance is necessary to cover sporadic, human-to-human, foodborne, and waterborne outbreaks. The timeliness of norovirus outbreaks report in China has improved in recent years, facilitating timely control of the outbreaks.

Norovirus is highly contagious and mainly spreads through the fecal-oral route (24). People can become infected through close contact with infected individuals or by contaminated food, water, or aerosols. Waterborne and foodborne outbreaks are more likely to result in larger-scale infections and outbreaks within populations (30–32). While there are still reports of small-scale outbreaks caused by aerosol transmission (22, 33). During the early stages of Norovirus infection, vomitus can contain a high viral load, facilitating its spread in enclosed dormitories, public spaces, and on surfaces of public objects (24, 34), aerosols which are easily formed can be inhaled, leading to infection (33, 35, 36). Studies in the United States, Germany, and Australia have demonstrated that the outbreak and detection rate of norovirus infection have decreased significantly through non-pharmaceutical interventions such as closing schools, restaurants, and other public places, promoting social distancing, and raising personal hygiene awareness since the COVID-19 outbreak in 2020 (37–39). The norovirus detection rate in China in 2020 was 58.76% lower than the average from 2012 to 2019 (40). It is evident that closing public places and minimizing gatherings of people are essential to reduce the incidence of norovirus.

The index case vomited in the classroom, but the pre-school teacher did not promptly evacuate the students. After a simple cleanup of the vomitus, no disinfection was conducted, and the vomitus was left in the classroom trash can. Additionally, as it is currently winter, the coldest month in Chongqing, the teaching buildings are less ventilated and relatively enclosed, increasing the likelihood of early transmission through contact with or inhalation of virus aerosols suspended in the air. Seven suspected cases were seated around the index case, and two cases were near the trash can, indicating that they had more exposure to the vomitus or contaminants, which contributed to the interpersonal transmission of norovirus. Later in the outbreak, cases continued to vomit in the classroom, leading to the spread of infection, which became an important factor in the occurrence of this outbreak.

It is also important to consider the parents’ responsibility in this outbreak. The index case began vomiting on the way to kindergarten, while the parents did not inform the teacher about the situation, which shows that the inadequate attention to this kind of adverse event. Parents’ supervision of their children’s health should be strengthened, and any adverse symptoms should be reported to the school and the teacher immediately. As is common practice in school establishments, rules dictate that children with such symptoms should not be allowed into the premises.

Several control measures were implemented to quickly stop the outbreak. First, we conducted terminal disinfection of classrooms, toilets, and dormitory in the kindergarten using chlorine containing disinfectants (with an effective concentration of not less than 5,000 mg/L). Second, we suspended classes throughout the kindergarten to avoid further contact and gathering among students, which effectively reduces the spread of the epidemic (41). Third, all cases are required to stay at home, and they can return to school 3 days after the symptoms disappear; students without abnormalities can return to school 3 days after the suspension of classes. Fourth, training has been conducted for all principals and teachers responsible for safety in the entire Rongchang. The training includes how to properly handle vomitus, how to disinfect environments that may be contaminated by the virus, and how to quickly establish a student health reporting system during the norovirus epidemic season.

The current study emphasizes the correct handling and disinfection methods for norovirus vomitus, which are crucial in preventing the outbreak and spread of norovirus. Therefore, we suggest the following procedure for the proper handling of vomitus from norovirus cases (33, 42, 43): (1) Evacuate people around the vomitus, If in a closed space, windows should be opened to improve ventilation and reduce the concentration of virus aerosols in the air, which can help prevent the spread of norovirus. (2) Personnel should wear protective gear for handling vomitus, including a cap, two pairs of gloves, a mask, and shoe covers. Etc. This equipment is essential for preventing direct contact with the vomitus and reducing the risk of contamination. (3) Use disposable absorbent materials such as gauze or cloth, soaked completely in a disinfectant solution (with an effective chlorine content of not less than 5,000 mg/L), to cover the entire surface of the vomitus. Carefully remove the absorbent material and place it in a yellow garbage bag or special trash can containing disinfectant solution (with an effective chlorine content of not less than 5,000 mg/L) for 30 min as medical waste, then dispose of it. (4) Disinfect gloves with 5% iodophor cotton, remove outer gloves and protective equipment, then remove the next pair of gloves, and thoroughly clean hands. (5) For vomitus or diarrhea in toilets or latrines, first evenly sprinkle chlorine-containing disinfectant powder (such as bleach) on the surface (including the surroundings) for coverage, cover the toilet lid, rinse with water after 30 min. (6) After the vomitus is cleared, thoroughly disinfect the environment contaminated by the vomitus and the cases. Wipe and disinfect the surfaces of objects such as floors, tables, chairs, and walls contaminated by vomitus with disinfectant solution (with an effective chlorine content of not less than 1,000 mg/L) or disinfectant wipes from the emergency disposal pack for contaminated materials. The disinfection range is 2 meters around the vomitus, and it is recommended to wipe twice. After 30 min of disinfection, wipe (mop) clean with water. (7) Conduct a comprehensive cleaning and disinfection of other public areas in the unit within 24 h.

This study emphasizes the need for better handling of vomitus containing norovirus and appropriate disinfectant methods to address the outbreak of norovirus acute gastroenteritis. In order to better prevent and control norovirus, some key suggestions in norovirus infection control are recommended: (1) raise awareness rate of norovirus among students and staff. Students are requested to promptly report suspected norovirus infection symptoms such as diarrhea and vomiting, and schools should isolate cases promptly. (2) It is recommended that a designated person be responsible for handling vomitus, excrement, and contaminated environmental hygiene. (3) Students should avoid direct or close contact with patients and their vomitus. (4) Schools and childcare institutions should strengthen daily morning and afternoon inspections and tracking of absences due to illness to promptly detect and isolate cases. (5) Implement class suspension measures promptly. (6) Medical institutions and disease control agencies should strengthen cooperation in monitoring NoV cases, conduct timely whole-genome sequencing and molecular epidemiological analysis of obtained strains, especially those in cluster and outbreak situations, accurately assess the local strain epidemic situation, and provide scientific basis for locally targeted epidemic prevention and control strategies.

The current study has several limitations. First, in this outbreak, genetic sequencing of norovirus was not conducted to determine which genotype of norovirus was involved. However, due to the large number of cases that occurred in a short period, they may have been affected by the same strain. Second, due to limited time and manpower, asymptomatic students were not tested for norovirus by collecting anal swabs. Third, there was a 2 days delay between the onset of symptoms and the epidemiological investigation, which may have led to recall bias. However, we corrected any biases by strengthening interviews with staff and reviewing surveillance videos. At last, the outbreak reported to RCCDC was late at night, so it was not possible to collect the food provided in kindergarten for norovirus detection. However, anal swabs of the chef and surface swabs in the kitchen were obtained to help rule out the possibility that food may be not a risk factor for the current norovirus outbreak.

## 5 Conclusion

Evidence from epidemiologic surveys and nucleic acid detection for norovirus suggests that the outbreak was primarily caused by improper disposal of vomitus and that close contact between kindergarten students accelerated the spread of norovirus. Outbreaks of norovirus can be effectively prevented if the vomitus and environment are handled and disinfected in a standardized manner by well-trained school personnel. A proactive surveillance program of endemic noroviruses remains essential to the detection, prevention, and control of future viral diarrhea outbreaks and moreover, the need for the advancement of ongoing vaccine development efforts.

## Data availability statement

The raw data supporting the conclusions of this article will be made available by the authors, without undue reservation.

## Ethics statement

The studies involving humans were approved by this study had been approved by the Ethics Committee of Rongchang Center for Disease Control and Prevention (NO. RCJK20230023). The studies were conducted in accordance with the local legislation and institutional requirements. Written informed consent for participation in this study was provided by the participants' legal guardians/next of kin. Written informed consent was obtained from the individual(s), and minor(s)' legal guardian/next of kin, for the publication of any potentially identifiable images or data included in this article.

## Author contributions

HX: Data curation, Formal analysis, Methodology, Project administration, Writing – original draft, Writing – review & editing. FM: Conceptualization, Formal analysis, Methodology, Validation, Writing – review & editing. DT: Investigation, Software, Writing – original draft. DL: Resources, Validation, Writing – review & editing.
